# The process-related dynamics of microbial community during a simulated fermentation of Chinese strong-flavored liquor

**DOI:** 10.1186/s12866-017-1106-3

**Published:** 2017-09-15

**Authors:** Yanyan Zhang, Xiaoyu Zhu, Xiangzhen Li, Yong Tao, Jia Jia, Xiaohong He

**Affiliations:** 1 Key Laboratory of Environmental and Applied Microbiology, Chinese Academy of Sciences & Environmental Microbiology Key Laboratory of Sichuan Province, Chengdu Institute of Biology, Chinese Academy of Sciences, Chengdu, 610041 People’s Republic of China; 2 University of Chinese Academy of Sciences, Beijing, 100049 People’s Republic of China; 3 Chengdu Institute of Biology, Chinese Academy of Sciences, Chengdu, 610041 People’s Republic of China

**Keywords:** Chinese strong-flavored liquor, Microbial community, Dynamics, Flavoring chemicals

## Abstract

**Background:**

Famous Chinese strong-flavored liquor (CSFL) is brewed by microbial consortia in a special fermentation pit (FT). However, the fermentation process was not fully understood owing to the complicate community structure and metabolism. In this study, the process-related dynamics of microbial communities and main flavor compounds during the 70-day fermentation process were investigated in a simulated fermentation system.

**Results:**

A three-phase model was proposed to characterize the process of the CSFL fermentation. (i) In the early fermentation period (1–23 days), glucose was produced from macromolecular carbohydrates (e.g., starch). The prokaryotic diversity decreased significantly. The *Lactobacillaceae* gradually predominated in the prokaryotic community. In contrast, the eukaryotic diversity rose remarkably in this stage. *Thermoascus*, *Aspergillus*, *Rhizopus* and unidentified *Saccharomycetales* were dominant eukaryotic members. (ii) In the middle fermentation period (23–48 days), glucose concentration decreased while lactate acid and ethanol increased significantly. Prokaryotic community was almost dominated by the *Lactobacillus*, while eukaryotic community was mainly comprised of *Thermoascus*, *Emericella* and *Aspergillus*. (iii) In the later fermentation period (48–70 days), the concentrations of ethyl esters, especially ethyl caproate, increased remarkably.

**Conclusions:**

The CSFL fermentation could undergo three stages: saccharification, glycolysis and esterification. *Saccharomycetales*, *Monascus*, and *Rhizopus* were positively correlated to glucose concentration (*P* < 0.05), highlighting their important roles in the starch saccharification. The *Lactobacillaceae*, *Bacilli*, *Botryotinia*, *Aspergillus*, unidentified *Pleosporales* and *Capnodiales* contributed to the glycolysis and esterification, because they were positively correlated to most organic acids and ethyl esters (*P* < 0.05). Additionally, four genera, including *Emericella, Suillus, Mortierella* and *Botryotinia*, that likely played key roles in fermentation, were observed firstly. This study observed comprehensive dynamics of microbial communities during the CSFL fermentation, and it further revealed the correlations between some crucial microorganisms and flavoring chemicals (FCs). The results from this study help to design effective strategies to manipulate microbial consortia for fermentation process optimization in the CSFL brew practice.

**Electronic supplementary material:**

The online version of this article (10.1186/s12866-017-1106-3) contains supplementary material, which is available to authorized users.

## Background

Chinese strong-flavored liquor (CSFL) is a typical representatives of Chinese liquor, accounting for about 70% of Chinese liquor market share [1]. The CSFL is produced by the Chinese classic solid-state fermentation, which involves a spontaneous process with simultaneous saccharification and fermentation [2]. The procedure details include mixing pre-culture starter (Daqu) [3] and pulverizing grains (e.g., sorghum, corn, wheat and rice) [4], filling the mixture into the fermentation pit (FT, hereafter, unless otherwise indicated) under the ground and sealing it with mud (Additional file 1: Figure S1). Daqu is a traditional fermentation starter, which is produced in an open environment from non-sterilized raw materials, e.g. raw wheat, barley and/or pea. It is reported that *Lactobacillus*, *Bacillus*, *Aspergillus*, and some non-*Saccharomyces* genera (*Saccharomycopsis*, *Pichia*) are dominant microbes in different types of Daqu [3]. After fermenting for 60–70 days, the fermented grains (also called Zaopei) are taken out from the FT and are mixed a number of fresh pulverizing grains,and are distilled to gain the CSFL. After that, the steamed grains (a mixture of Zaopei and fresh grains) are reused by mixing Daqu for fermentation again [1, 5]. The microorganisms play critical roles for the production of the CSFL because they can convert carbohydrates (e.g., starch, sucrose and glucose) into ethanol [4, 6–9]. In addition, microbes also produce various flavoring compounds, such as lactic acid, butyric acid, caproic acid, and ethyl caproate [10–12]. In particular, caproic acid and ethyl caproate are defining flavoring substances that determines the quality of the CSFL to a large degree [1].

High CSFL quality is attributed to the dynamics of microbial community and their metabolisms in the fermentation process. Previously, the CSFL fermentation microbiota have been studied using cultivation-dependent and -independent approaches using denaturing gradient gel electrophoresis (DGGE) and clone library analysis of the 16S rRNA gene [4, 13, 14]. However, big discrepancies in microbial compositions existed among previous investigations. This may be attributable to differences in sampling time (different stages of fermentation), and laboratory techniques employed to characterize the community structure. In addition, most of the previous studies on the CSFL fermentation microbiota using traditional cultural and molecular methods cannot provide details of the phylogenetic compositions and process-related changes of microbial community [4, 9, 13, 15–17]. However, understanding the process-related dynamics of microbial community is important to design effective strategies to manipulate microbial consortia for fermentation process optimization in the CSFL brew practice. The next generation sequencing technique provided powerful tools to reveal the microbial community dynamics in the complicated environments [6].

In this study, we investigated the process-related dynamic of microbial communities (bacteria, archaea and fungi) and metabolites during different fermentation stages (1, 10, 23, 34, 48, 59, and 70 days) using MiSeq-sequencing targeting 16S rRNA and ITS genes, respectively, and identify the correlations between key microbial taxa and the flavoring compounds of the CSFL.

## Methods

### Sampling

The production of CSFL undergoes anaerobic fermentation in the FT under the ground. Sampling from real fermentation pit would disrupt fermentation process. Thus, we used batch experiments to simulate CSFL fermentation using glass bottles of 3.5 L (Additional file 1: Figure S2) as laboratory reactors. The fermentative samples (a mixture of Zaopei, Daqu, and fresh grains including sorghum, corn, wheat and rice, same as the real sample in the distiller) were collected from a well-known distillery, located in Mianzhu city, Sichuan province, China. A total of 21 bottles were filled with the fermentative samples described above, and sealed with mud,frosted-glass stopper and plastic sheets (Additional file 1: Figure S2), and cultivated at 30 °C for 70 days. During different fermentation stages of 1, 10, 23, 34, 48, 59, and 70 days, three parallel bottles were sacrificed for sampling each time. Samples were stored at −80 °C for the further use.

### Chemical and physical property analysis

The pH was measured using pH meter in the suspension liquid after sample centrifugation, with a 1:5 ratio of sample to deionized water. The moisture was measured using a gravimetric approach by drying samples between 103 °C–105 °C for 48 h after sampling. For the detection of organic acid, such as lactic acid, acetic acid, butyric acid, and caproic acid, as well as glucose and ethanol content, 5 g of sample was vortex mixed with 25 mL of deionized water, centrifuged and filtered through a 0.22 μm MCE filter, and the metabolite contents were quantified using HPLC (Agilent 1260,USA). The operating condition of HPLC was as follows: Hi-Plex H HPLC column (300 × 6.5 mm), refractive index detector (RID detector), 5 mM H_2_SO_4_ as mobile phase with the velocity of 0.6 mL/min, and column temperature was 55 °C. For the detection of esters, such as ethyl acetate, ethyl caproate, and ethyl lactate, 5 g of sample was vortex mixed with 25 mL of ethanol, centrifuged and filtered through a 0.22 μm filter, and esters contents were determined with GC (Agilent 7890A,USA). The operating condition of GC was as following: Agilent DB-WAX column (30 m × 530 μm × 1 μm), fame ionization detector (FID detector), 40 mL/min of H_2_ flow rate, 300 mL/min of air flow rate, and N_2_ as the carrier gas with the velocity of 15 mL/min.

### DNA extraction, PCR amplification and MiSeq sequencing

The genomic DNA was extracted from a total of 21 samples taken from seven fermentation stages using Soil DNA Kit (Omega Bio-tek, Inc.) following the manufacturer’s protocol. The DNA quality and quantity were determined by NanoDrop 2000 (Thermo, USA). For prokaryotes, the V4 hypervariable region of the 16S rRNA genes was amplified using universal primer 515F and 909R [18]. For eukaryotes, the ITS2 region of fungal rRNA gene was amplified using universal primer ITS4 and ITS7 [19]. Primer 515F and ITS4 were added with barcodes. PCR conditions were described in detail previously [20]. The amplified PCR products were analyzed through a 1%(wt/vol) agarose gel and purified using a PCR purification kit (GE0101–50, TSINGKE). The concentrations of PCR purified products were assessed by NanoDrop 2000 (Thermo, USA). Subsequently, purified amplicons of all samples were equally pooled for constructing a PCR amplicon library, according to the protocols of the Illumina TruSeq. DNA sample preparation LT kit (San Diego, CA, USA), and then subjected to sequencing using the Illumina MiSeq platform at the Environmental Genomic Platform of the Chengdu Institute of Biology, CAS.

### Sequencing data analysis

Sequencing data analysis was performed by QIIME Pipeline Version 1.7.0 [21]. The raw sequences were sorted with their unique barcodes. Sequences with low quality, read length below 200 bp as well as average base quality score less than 30, were filtered out. Chimera sequences were removed utilizing Uchime algorithm [22].

Sequences were clustered into operational taxonomic units (OTUs) at a 97% identity threshold. Each sample was rarefied to the same number of reads (10,568 reads for 16S rRNA gene and 4937 reads for ITS gene, respectively) for both alpha-diversity (chao1 estimator of richness, observed species and Shannon’s index) and beta-diversity (PCoA, UniFrac) analyses. Taxonomy was assigned using the Ribosomal Database Project classifier (http://rdp.cme.msu.edu/).

### Statistical analysis

The changes of microbial community during fermentation were evaluated by principal coordinates analysis (PCoA, UniFrac). The PerMANOVA was performed with R to present the statistical significance among datasets based on the weighted PCoA scores. One-way analysis of variance (ANOVA) was conducted to compare the differences of microbial communities among intra-group and inter-groups. Pearson’s correlation analysis was performed to determine the correlations between variables. Phylogenetic analysis (maximum likelihood algorithm) of OTUs with reference sequences was performed using MEGA6 version 6 [23]. Canonical correspondence analysis (CCA) was conducted using CANOCO 5.0 (Microcomputer Power, Ithaca, NY) to confirm the correlations between community structures and environmental variables.

### Nucleotide sequence accession number

The original sequencing data are available at the European Nucleotide Archive at accession no. PRJEB19772 (http://www.ebi.ac.uk/ena/data/view/PRJEB19772).

## Results

### Chemical and physical properties during fermentation

Chemical and physical properties of Zaopei at different fermentation stages were shown in Table 1. During the early stage (1–23 days), glucose increased quickly, and reached a peak of 28.43 mg/g on day 23. During this period, no organic acids and ethyl esters were produced, and pH (pH 3.4) maintained constant. Ethanol concentration began to increase slightly. During the middle stage (23–48 days), glucose declined sharply. Whereas, lactic acid and ethanol began to be produced constantly. Acetic acid, propionic acid and ethyl esters changed little. The pH decreased to 3.2 with the increase of lactic acid production. During the late stages (48–70 days), glucose decreased further. Lactic acid and ethanol concentrations increased clearly, and reached up to 36.87 mg/g and 9.25 mg/g on day 70 (Table 1). Considerably, the ethyl lactate (876.82 μg/g), ethyl acetate (138.85 μg/g) and ethyl caproate (96.14 μg/g) were produced significantly at the end of fermentation (Table 1).

**Table 1:** Chemical and physical properties of samples at different fermentation stages

Sample time (day)	1	10	23	34	48	59	70
pH	3.39 ± 0.01^a^	3.40 ± 0.01^a^	3.42 ± 0.03^a^	3.35 ± 0.04^ab^	3.25 ± 0.02^bc^	3.21 ± 0.04^c^	3.24 ± 0.01^c^
Moisture (%)	56.78 ± 0.00^a^	56.02 ± 0.00^a^	57.25 ± 0.00^a^	57.67 ± 0.01^a^	58.58 ± 0.01^bc^	58.57 ± 0.00^bc^	60.23 ± 0.02^c^
Glucose (mg/g)	7.27 ± 1.14^a^	19.68 ± 6.60^b^	28.43 ± 0.81^c^	17.17 ± 2.57^b^	6.73 ± 1.15^a^	4.58 ± 0.36^a^	5.62 ± 1.13^a^
Lactic acid (mg/g)	22.23 ± 0.76^a^	20.59 ± 1.31^a^	21.84 ± 1.27^a^	27.15 ± 1.41^b^	29.39 ± 1.12^b^	34.75 ± 0.22^c^	36.87 ± 1.13^c^
Acetic acid (mg/g)	1.27 ± 0.03^a^	1.20 ± 0.08^a^	1.36 ± 0.11^a^	1.68 ± 0.15^b^	2.16 ± 0.06^c^	2.61 ± 0.05^d^	2.73 ± 0.32^d^
Propanic acid (mg/g)	3.68 ± 0.06^a^	3.41 ± 0.02^b^	3.61 ± 0.12^ab^	3.56 ± 0.11^ab^	4.51 ± 0.22^c^	4.45 ± 0.14^c^	4.56 ± 0.09^c^
Ethanol (mg/g)	0.28 ± 0.16^a^	0.54 ± 0.23^a^	2.00 ± 0.68^ab^	3.84 ± 1.38^b^	5.80 ± 0.17^bc^	7.65 ± 0.40^c^	9.25 ± 2.63^c^
Ethyl acetate (ug/g)	56.9 ± 2.42^a^	59.5 ± 3.74^ab^	70.7 ± 4.10^b^	79.7 ± 9.37^b^	92.1 ± 1.66^b^	127.8 ± 10.20^c^	138.8 ± 14.24^c^
Ethyl caproate (ug/g)	ND*	ND	ND	ND	ND	93.3 ± 5.64^a^	96.1 ± 5.80^a^
Ethyl lactate (ug/g)	441.6 ± 32.96^a^	358.6 ± 18.37^a^	400.9 ± 28.31^a^	41.72 ± 69.04^a^	481.8 ± 39.85^a^	730.25 ± 25.42^b^	876.82 ± 99.47^c^

*ND: not detected. All data are presented as means ±standard deviations (*n* = 3). Values with different letters in a row mean significant differences at *P* < 0.05 determined by ANOVA

### Microbial community structure and diversity

**Fig. 1:**
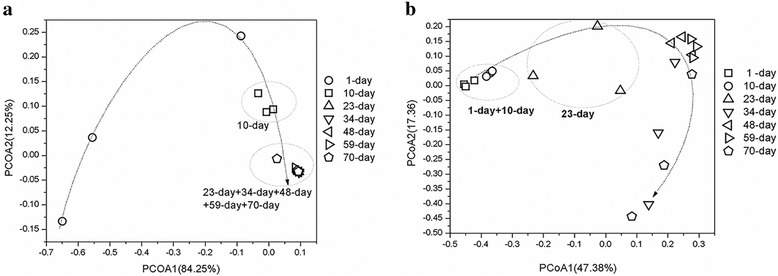
Principal coordinates analysis (PCoA) of overall microbial communities at different fermentation stages. **a** prokaryotic community; **b** eukaryotic community. Different colors represented different fermentation time and each sampling had three replicates

For 16S rRNA gene sequences, we resample to 10,568 reads per sample. The rarefaction curves reached the saturation plateau and the Good’s coverages among samples were more than 95% (Additional file 2: Table S1, and Additional file 1: Figure S3). The OTU numbers of samples ranged from 192 to 976 based on the cutoff of 97% identity. As shown in Table 2, the Shannon diversity index and Chao1 estimator of richness during 1–10 days were significantly higher than those at other stages (*P* < 0.05, Table 2). PCoA analysis based on weighted UniFrac method showed that there were three clusters (Fig. 1a). The 1-day samples scattered randomly, while the 10-day samples closely clustered together. The samples from 23 to 70 days formed another cluster. PerMANOVA analysis demonstrated that there were no significant differences among prokaryotic communities of 23 to 70-day samples (*p* > 0.05), but significant different from prokaryotic communities in early days (1 to 10-day).

**Table 2:** Microbial diversity indices calculated based on the cutoff of 97% identity of 16S rRNA gene or ITS region

Sample time (day)	Chao1		Observed species		Shannon index	
	16S rRNA gene	ITS gene	16S rRNA gene	ITS gene	16S rRNA gene	ITS gene
1	1462 ± 155^a^	278.27 ± 52.17^a^	976 ± 84^a^	170 ± 12^a^	7.04 ± 0.26^a^	3.84 ± 0.25^a^
10	1414 ± 248^a^	346.14 ± 21.58^ab^	789 ± 54^b^	197 ± 8^ab^	5.49 ± 0.19^b^	3.99 ± 0.11^ab^
23	566 ± 78^b^	609.34 ± 214.43^b^	212 ± 9^c^	343 ± 136^b^	2.56 ± 0.07^c^	5.17 ± 1.04^bc^
34	683 ± 138^b^	825.28 ± 230.83^c^	192 ± 4^c^	479 ± 100^c^	2.52 ± 0.04^c^	6.14 ± 1.01^c^
48	559 ± 119^b^	498.68 ± 125.07^ab^	198 ± 7^c^	276 ± 62^ab^	2.56 ± 0.06^c^	4.05 ± 0.57^ab^
59	631 ± 97^b^	560.67 ± 24.61^c^	239 ± 5^c^	308 ± 34^ab^	2.70 ± 0.07^c^	4.33 ± 0.31^ab^
70	784 ± 270^b^	777.76 ± 218.55^c^	333 ± 200^c^	462 ± 106^c^	3.07 ± 0.83^c^	5.96 ± 0.81^c^

*All data were presented as means ± standard deviations. Values with different letters in a column mean significant difference at *p* < 0.05 tested by one-way ANOVA Duncan’s test

For eukaryotic community, 157 to 582 OTUs were observed for all samples based on 97% similarity as a cutoff (Table 2). The Good’s coverages among samples were more than 90% (Additional file 2: Table S2). In contrast to the prokaryotic community, eukaryotic Chao 1 estimator, Shannon index and observed OTU numbers firstly increased, then fluctuated with the fermentation process. During the whole fermentation, the succession of the eukaryotic communities was slightly different from that of prokaryotic community. PCoA analysis showed samples from 1 to 10 days formed a cluster, and samples from 23rd day formed another cluster. Samples from 48 to 59 days clustered together again. However, samples from 70th day scattered (Fig. 1b).

### Microbial community compositions

**Fig. 2:**
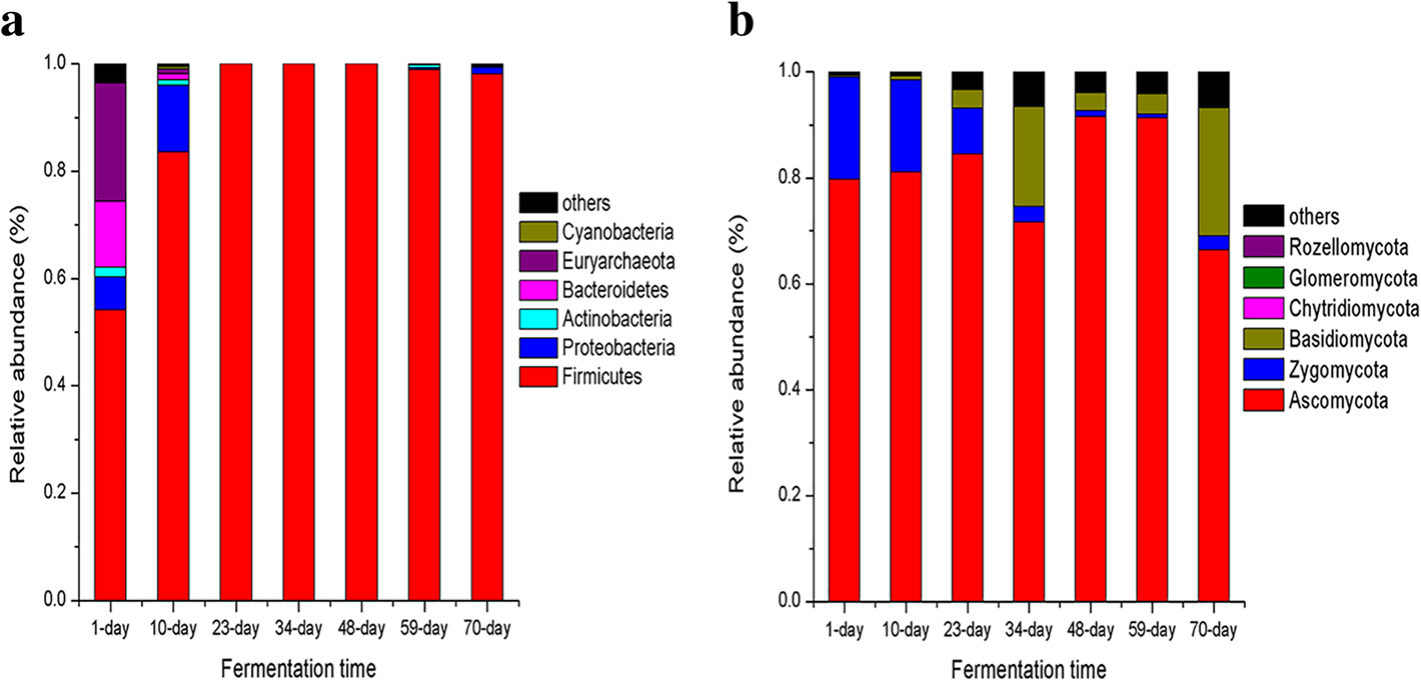
Relative abundance plots of microbial community composition during the entire fermentation period at phylum level. **a** prokaryotic community at phylum level, (**b**) eukaryotic community. Each value was the mean of triplicate samples

At phylum level, there were six dominant prokaryotic phyla observed throughout whole fermentation process: *Firmicutes, Proteobacteria, Actinobacteria, Bacteroidetes, Euryarchaeota* and *Cyanobacteria. Firmicutes* was the most abundant phylum, accounting for average 54.2% to 99.9% of the total prokaryotic community during the whole fermentation process. During 1–10 days, *Firmicutes* abundance were 54.19 to 83.61% of the whole communities, and all other phyla approximately occupied 45.81% to 16.49% of the microbiota, including *Proteobacteria* (6.15–12.35%), *Actinobacteria* (1.73–0.97%), *Bacteroidetes* (12.28–1.17%), *Euryarchaeota* (21.99–0.89%). As fermentation proceeded, the *Firmicutes* abundance significantly increased (*P* < 0.05) up to more than 99% during 23 and 70 days (Fig. 2a). a large proportion (>90%) of prokaryotic reads failed to be classified to the genus level. The sequence analysis on OTU level was carried out by NCBI BLAST server and RDP CLASSIFIER. As shown on Additional file 2: Table S2, prokaryotic community was dominated by eight OTUs including OTU17 (23.98–45.19% of total 16S rRNA reads), OTU27 (16.46–28.07%), OTU 211 (5.81–8.64%), OTU 125 (3.56–6.40), OTU 4 (1.51–1.78%), OTU 218 (1.15–1.38%), OTU 216 (0.0–1.53%), OTU 48 (0.0–1.23%). These OTUs accounted for 60.8–93.4% of prokaryotic community. Most of these OTUs showed high similarity (>95%) with uncultured Lactobacillus sp. clone 16S ribosomal RNA gene, but low similarity (<93%) with isolates (members of genus Lactobacillus) in NCBI’s GenBank. Moreover, these OTU reads could not be classified to the genus level by RDP CLASSIFIER. Therefore, community composition was analyzed at family level. A total of 17 abundant families (abundance > 1%) were detected. Among them, 11 families were affiliated to phylum *Firmicutes*, including *Lactobacillaceae, Ruminococcaceae, Tissierellaceae, Bacillaceae, Clostridiaceae, Syntrophomonadaceae, Planococcaceae,* unclassified *Bacilli, Leuconostocaceae, Streptococcaceae*, and unclassified *Lactobacillales.* In particular, *Lactobacillaceae* and unclassified *Bacilli* almost dominated the microbiota during the middle and later fermentation stages (Fig. 4a). Three families were affiliated to *Proteobacteria*, including *Xanthomonadaceae, Pseudomonadaceae* and *Moraxellaceae*; Two families were affiliated to Euryarchaeota, including *Methanosarcinaceae* and *Methanobacteriaceae*; One family was affiliated to *Porphyromonadaceae* (phylum *Bacteroidetes*). To figure out the compositions and succession of microbial community more specifically, a heatmap of prokaryotic OTUs was performed (Additional file 1: Figure S4). We identified 27 representative OTUs (abundance >1%). Only two OTUs (OTU17, OTU27) were shared by all samples with their abundances from 3.69% to 45.63%. Both of them increased rapidly on 23rd day followed by stabilizing generally in the mid-late period. The OTU17 and OTU27 revealed low similarities with their closest phylogenetic neighbour (members of genus Lactobacillus, Additional file 2: Table S2). A large proportion of diverse OTUs just appeared in early period, however most of them decreased quickly in the mid-late period and remained in a low abundance. After day 23, the entire prokaryotic population was almost covered by 8 OTUs (OTU17, OTU27, OTU 211, OTU 125, OTU 4, OTU 218, OTU 216, and OTU 48), all of which were affiliated to *Lactobacillaceae* (Additional file 2: Table S3, and Additional file 1: Figure S5).

**Fig. 3:**
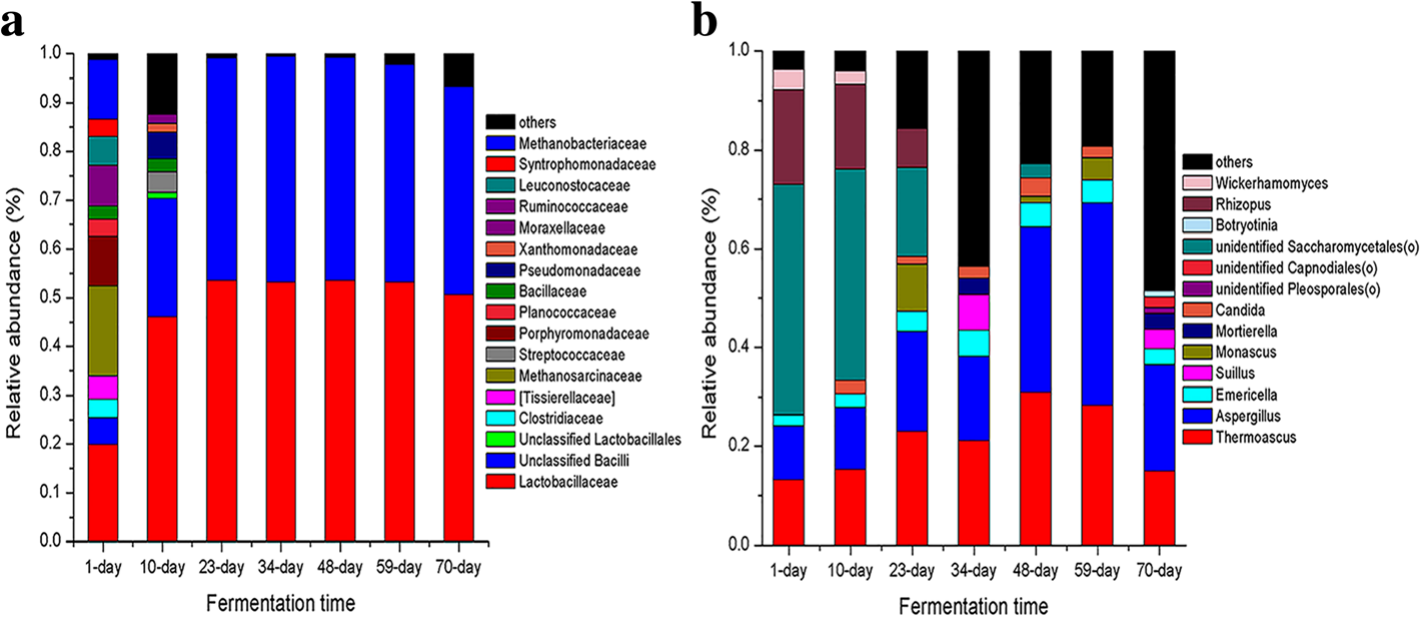
Relative abundance plots of microbial community composition during the entire fermentation period. **a** prokaryotic community at family level, (**b**) eukaryotic community at genus level. Each value was the mean of triplicate samples

Six phyla were observed in eukaryotic community, including *Ascomycota, Zygomycota, Basidiomycota, Chytridiomycota, Glomeromycota* and *Rozellomycota.* Among them, *Ascomycota, Zygomycota and Basidiomycota* occurred throughout the entire fermentation process, while Ascomycota predominated at the average relative abundance of 80.9%. During the early stages (1 to 10-day), *Zygomycota* was a subdominant group (17.36–19.26%), but it sharply decreased with the fermentation process up to 2.61% on the 70th day. In contrast, *Basidiomycota* abundance increased from 0.33% to 24.27% during the fermentation (Fig. 2b). At genus level, 13 abundant genera (abundance >1%) affiliated to three phyla were observed, including ten *Ascomycota* genera (*Thermoascus, Aspergillus, Emericella, Monascus, Candida,* unidentified *Pleosporales, unidentified Capnodiales, unidentified Saccharomycetales, Botryotinia* and *Pichia*), one genera (*Suillus*) belonging to *Basidiomycota*, and two genera (*Mortierella, Rhizopus*) belonging to *Zygomycota.* The *Thermoascus*, *Aspergillus* and *Emericella* were defined as the core genera because of their presence in whole stage, especially *Thermoascus* and *Aspergillus* with their abundances over 10% on average (Fig. 3b). Further, 16 dominant OTUs (abundance >1%) were observed. The OTU 130 and OTU 6, affiliated to *Candida* and *Aspergillus*, respectively, were shared by all the samples with abundances from 27.07% to 57.98%. The OTU 130 that was closely related to *Candida humilis*, dominated the entire process accounting for an average of 22.15% (Additional file 2: Table S4). Remarkably, three OTUs (OTU 2056, OTU 756, and OTU 4385) only appeared in late periods (59 to 70-day), and they were affiliated to *Botrytis, Mortierella* and *Cladophialophora*, respectively (Additional file 2: Table S4 and Additional file 1: Figure S6).

### Correlations between microbial communities and flavoring chemicals

**Fig. 4:**
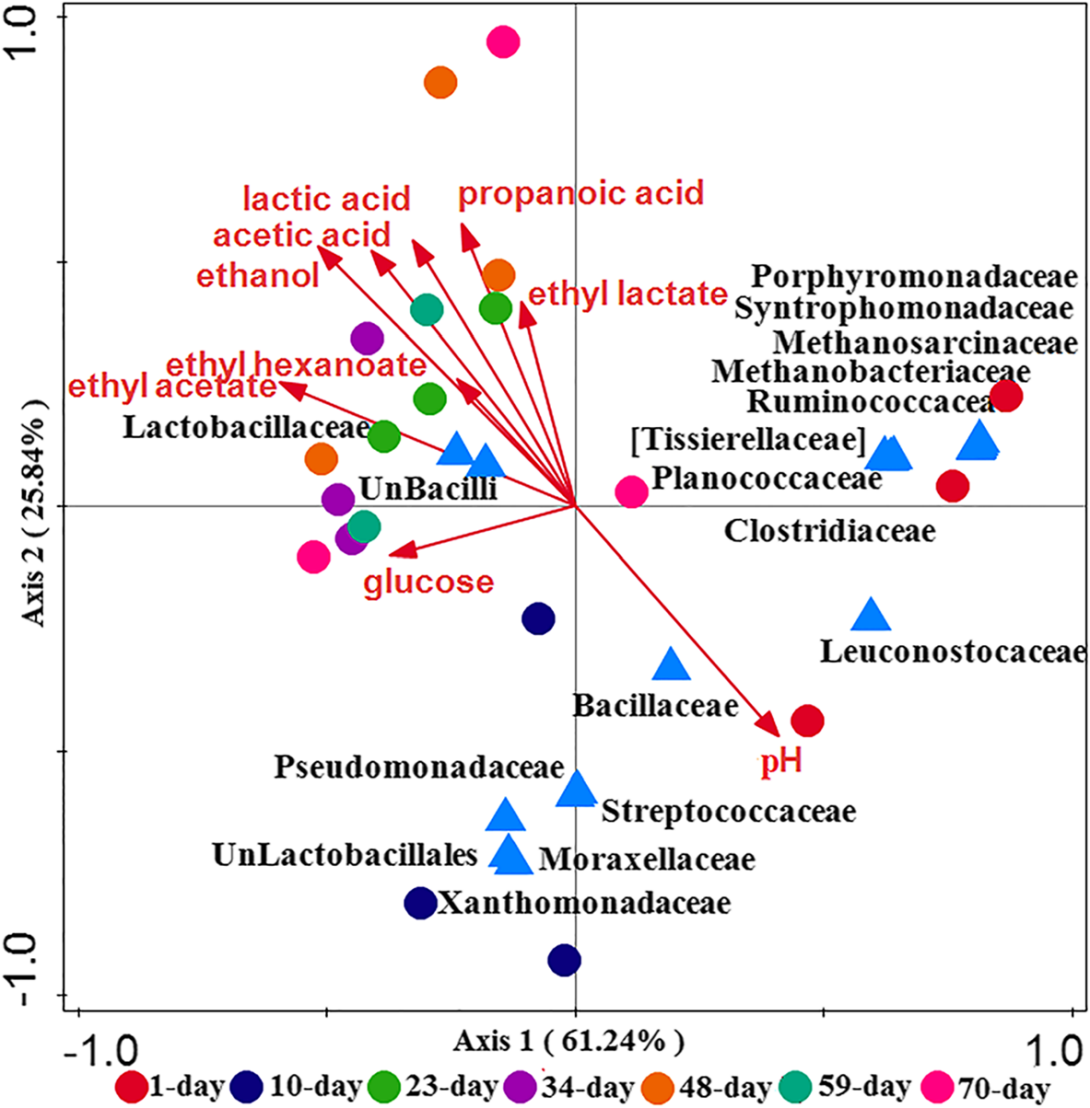
Canonical correspondence analysis (CCA) of prokaryotic community and flavoring chemicals. The circles with different colors represented microbial communities at different fermentation stages. The triangles in blue represented prokaryotic microbes at family level

**Fig. 5:**
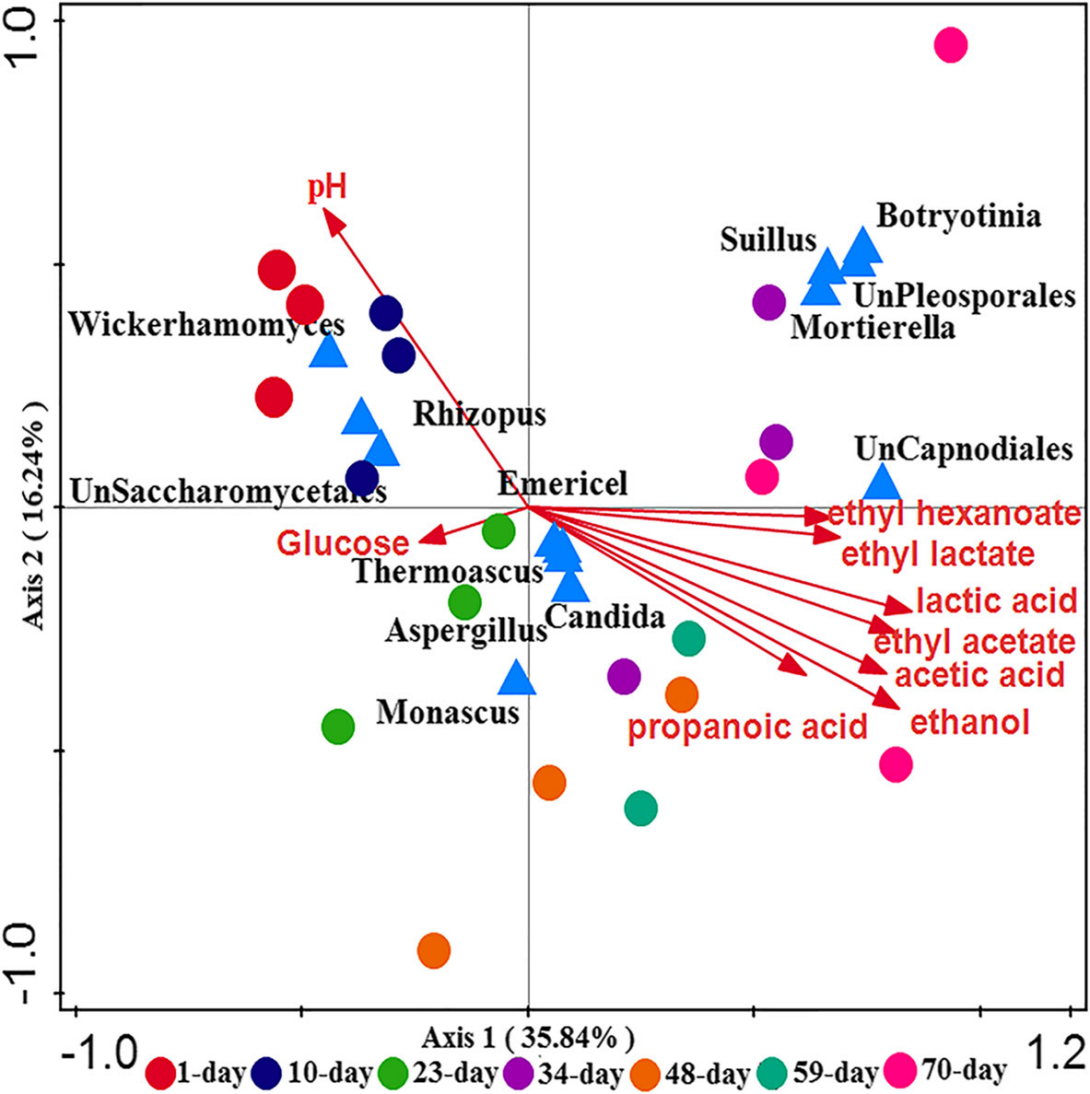
Canonical correspondence analysis (CCA) of eukaryotic community and flavoring chemicals. The circles in different colors represented microbial communities at different fermentation stages. The triangles in blue represented eukaryotic microbes at genus level

Canonical correspondence analysis (CCA) was conducted to reveal the correlations between microbial community and flavoring chemicals (FCs). For prokaryotes (Fig. 4), the first two axises explained 87.08% of the variation in community composition. The pH and ethanol content were the two most influential environmental variables. The organic acid and esters were key FCs that significantly correlated with communities during 23 to 70-day. The pH was closely correlated with the community composition with higher community diversity in early period (1 to 10-day). *Lactobacillaceae* and unclassified Bacilli were positively correlated with the FCs. At OTU level, eight prokaryotic members (OTU 4,OTU 48, OTU 17,OTU 27,OTU 211,OTU 218,OTU 125, and OTU 216) showed positive correlations with FCs (Additional file 1: Figure S7), especially ethyl acetate concentration (*p* < 0.05). These OTUs were assigned to *Lactobacillaceae* (Additional file 2: Table S3). For eukaryotes, both axes explained 52.08% of the variation in community composition, which is less than that in prokaryotic communities. Organic acid and esters levels were mainly positively correlated with eukaryotic communities in late period (34 to 70-day). The pH mainly correlated with the community composition in early period (1 to 10-day) with abundant unidentified *Saccharomycetales*, *Rhizopus* and *Pichia*. Figure 5 showed that unclassified *Capnodiales* was positively correlated with FCs (*p* < 0.01), and *Monascus* positively correlated with the production of glucose (*p* < 0.05). At OTU level, three eukaryotic OTU 756 (*Mortierella*), OTU 2056 (Botrytis), OUT 4385 (*Cladophialophora*) showed positive correlations with FCs (*p* < 0.01) (Additional file 2: Table S4, and Additional file 1: Figure S8).

## Discussion

Generally, the CSFL fermentation was achieved in strictly anaerobic FT under the ground, involving vastly complicated metabolic reactions and microbial community. It is difficult for sampling from underground pit during the fermentation process. Thus, microbial community in the FT is generally considered as a “black box”. Previous studies usually collect a limited number of samples at several points, or at starting point and end point [6, 13, 16, 17], which leads to the lack of understanding of the process-related dynamics of microbial community during the CSFL fermentation. In this study, the dynamics of microbial communities during CSFL fermentation was investigated by simulating fermentation using batch experiments to facilitate sampling. The final contents of lactic acid and ethanol in this study were similar to those of real fermentation process [24]. The main esters related with liquor’s quality were produced and the dynamics of microbial communities showed certain succession patterns. Notably, caproic acid and ethyl caproate are the important flavoring substances of CSFL, but caproic acid was not detected while ethyl caproate was detected until the late stages (59–70 days). Generally, caproic acid is mainly produced by microbes in Pit Mud (PM), while ethyl caproate is produced via the esterification by microorganisms mainly originated from Daqu starter in Zaopei [5, 25]. Compare to the real fermentation pit (Additional file 1: Figure S1), there was only a small number of PM in the bottom of in-vitro fermentation vessel (Additional file 1: Figure S2), and there was no PM on the wall inside the reactor. It was thus speculated that PM microbes were insufficient to produce detectable caproic acid in simulated fermentation, especially during the early and middle stage. During the late stage, PM microbes could produce a small amount of caproic acid that was esterified into ethyl caproate as soon as possible. Therefore, low concentration of ethyl caproate was detected on day 59 and 70, while caproic acid was not detected. To faithfully reproduce the real CSFL fermentation, more work is needed, such as the improvement of in-vitro fermentation vessel and preculture of Pit mud. Especially, It indicated that many novel microbes and their functions remain elusive.

The *Lactobacillaceae* and unclassified *Bacilli* were dominant and occurred throughout the entire fermentation, which are consistent with previous reports based on traditional molecular methods, e.g., DGGE, 16S rRNA gene clone library and PLFA [4, 26, 27]. The *Lactobacillaceae* could produce lactic acid from glucose or starch by homolactic fermentation [4]. Many members affiliated to *Bacilli* could produce various hydrolases for the liquefaction and saccharification of carbohydrates [26, 27]. Two core eukaryotic genera (*Aspergillus*, *Thermoascus*) existed in the entire fermentation process in our study. *Aspergillus* had the ability to produce various hydrolytic enzymes for starch saccharification, and *Thermoascus* could produce protease such as xylanase and α-amylase to degrade carbohydrate into sugars [27, 28]. Additionally, four new genera (*Emericella, Suillus, Mortierella and Botryotinia*) were observed in fermentation, which showed positive correlations with organic acid and ethyl esters (Fig. 5). Cao et al. [29] reported that *Emericella* existed in wheat Qu used for wheat Daqu fermentation. *Suillus* could decompose complex organic matter substrates, such as hemicellulose, cellulose and components of needles [30]. *Mortierella* was reported to have the ability to produce polyunsaturated fatty acid by degrading rice bran in solid substrate fermentation [31]. *Botryotinia* was reported to produce rhamnogalacturonan hydrolase, cell-wall-degrading enzymes and other low-molecular-weight compounds such as oxalic acid [32, 33]. However, their definite functions in CSFL fermentation still remain elusive. Ethyl esters are crucial factors that determine the quality of the CSFL [12]. Three microbial taxa (*Monascus, Candida and Pichia*) were reported to involve in the formation of ethyl esters [10, 17, 25, 34]. Microbial community showed distinct succession in the fermentation. In early fermentation period (1 to 23-day), the microbial communities were mainly dominated by *Lactobacillus* and eight eukaryotic genera (*Thermoascus, Aspergillus, Emericella, Monascus, Candida,* unidentified *Saccharomycetales, Rhizopus, and Pichia*). Glucoses were produced and reached a peak due to the degradation effects on macromolecular carbohydrates by above microbes [4, 6, 9, 13, 15, 17]. Meanwhile, to our knowledge, Daqu is an important saccharifying and fermenting agent [35]. Filamentous fungi (e.g. *Rhizopus, Aspergillus*), yeasts (e.g. *Saccharomyces, Candida*) and bacteria (e.g. acetic acid bacteria, lactic acid bacteria), are considered to be the functional populations in Daqu, which are responsible for lyase production and polysaccharide degradation [36]. Thus, this stage could be described as “the stage of saccharification”. In the middle fermentation period (23 to 48-day), *Lactobacillus* converts sugars into lactic acid, as the precursor to form ethyl lactate, and lead to the decrease of pH. Ethanol begins to be generated via the glycolysis by some bacterial and fungal microbes [7, 37, 38]. *Lactobacillus* absolutely predominates the microbial community, and eukaryotic community is dominated by *Thermoascus, Aspergillus, Emericella, Candida,* unidentified *Saccharomycetales*. During this period, lactic acid and ethanol are the main fermentation products. It could be described as “the stage of glycolysis stage”. At late fermentation period (48 to 70-day), ethyl esters are produced, especially ethyl caproate increases remarkably. In this stage, organic acids are transformed into esters by microbes, such as *Clostridium* and *Pichia* via esterification between alcohol and organic acids [9, 11, 36, 39]. Low pH results into the predominant of *Lactobacillus.* Eukaryotic populations fad significantly due to the unfavorable environmental conditions. The period could be described as “the stage of esterification”. The dynamics of microbial community in the CSFL fermentation process provide various metabolites which constitute unique flavor of Chinese liquor.

## Conclusion

This study comprehensively revealed a dynamic of microbial communities including prokaryotes and eukaryotes during the CSFL fermentation. The overall fermentation presents three phases: saccharification, glycolysis and esterification stage. During the fermentation, *Lactobacillaceae* was the most abundant prokaryotic taxon, and *Thermoascus, Aspergillus* and *Emericella* dominated entire eukaryotic communities during the whole fermentation. *Lactobacillaceae, Bacilli, Botryotinia*, *Aspergillus*, unidentified *Pleosporales* and *Capnodiales* were positively correlated to the production of FCs. *Emericella, Suillus, Mortierella* and *Botryotinia* were firstly observed in CSFL fermentation. This study provide deep theoretical basis to design effective strategies to manipulate microbial consortia for better controlling CSFL production systems and improving liquor quality in the brew practice.

## Additional files

Additional file 1: Figure S1. Schematic diagram of the real CSFL fermentation pit. **Figure S2.** Diagram of glass bottles used for simulating fermentation experiments. **Figure S3.** The rarefaction curves of sequencing depths. **Figure S4.** Heatmaps of prokaryotic and eukaryotic communities. **Figure S5.** Phylogenetic analysis (maximum likelihood algorithm) result of prokaryotic communities. **Figure S6.** Phylogenetic analysis (maximum likelihood algorithm) result of eukaryotic communities. **Figure S7.** Canonical correspondence analysis (CCA) of prokaryotic OTUs and flavoring chemicals. **Figure S8.** Canonical correspondence analysis (CCA) of eukaryotic OTUs and flavoring chemicals. (PDF 516 kb)

Additional file 2: Table S1. Good coverages of prokaryotic Sequencing (16S rRNA gene). **Table S2.** Good coverages of eukaryotic Sequencing (ITS gene). **Table S3.** The OTU BLAST result based on 16S rRNA gene. **Table S4.** The OTU BLAST result based on ITS region. (PDF 309 kb)

## Abbreviations

ANOVA: Analysis of variance
CCA: Canonical correspondence analysis
CSFL: Chinese strong-flavored liquor
FCs: Flavoring chemicals
FT: Fermentation pit
GC: gas chromatography
HPLC: High performance liquid chromatography
OTU: Operational taxonomic unit
PCoA: Principal coordinate analysis
PCR-DGGE: Polymerase chain reaction-denaturing gradient gel electrophoresis
PerMANOVA: Permutational multivariate analysis of variance
PLFA: Phospholipid fatty acid analysis
PM: Pit mud
qPCR: Real-time Quantitative polymerase chain reaction
RDP: Ribosomal Database Project
