# Supplementary material for: The process-related dynamics of microbial community during a simulated fermentation of Chinese strong-flavored liquor
Source: BMC Microbiol. 2017 Sep 15;17:196. doi: 10.1186/s12866-017-1106-3 (PMC5603089; doi:10.1186/s12866-017-1106-3)
Supplement: Supplementary file 1 — Schematic diagram of the real CSFL fermentation pit. Figure S2. Diagram of glass bottles used for simulating fermentation experiments. Figure S3. The rarefaction curves of sequencing depths. Figure S4. Heatmaps of prokaryotic and eukaryotic communities. Figure S5. Phylogenetic analysis (maximum likelihood algorithm) result of prokaryotic communities. Figure S6. Phylogenetic analysis (maximum likelihood algorithm) result of eukaryotic communities. Figure S7. Canonical correspondence analysis (CCA) of prokaryotic OTUs and flavoring chemicals. Figure S8. Canonical correspondence analysis (CCA) of eukaryotic OTUs and flavoring chemicals. (PDF 516 kb) [file 12866_2017_1106_MOESM1_ESM.pdf]

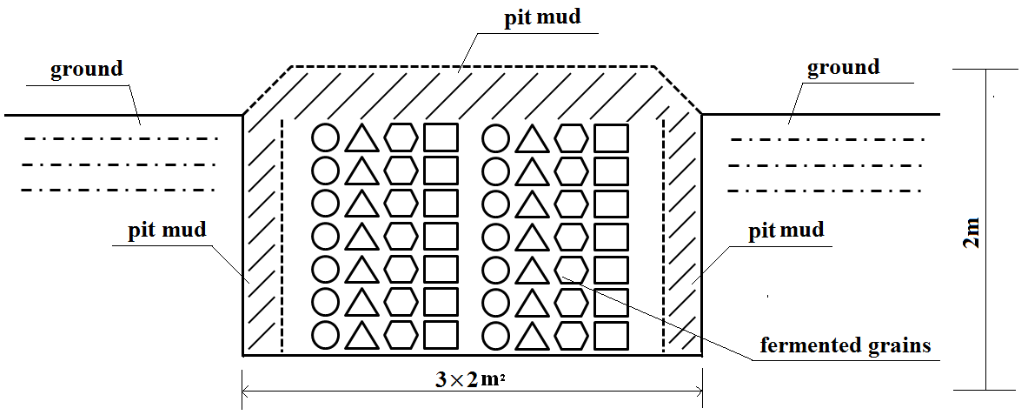

Fig. S1. Skeleton diagram of fermentation pit during the fermentation process.

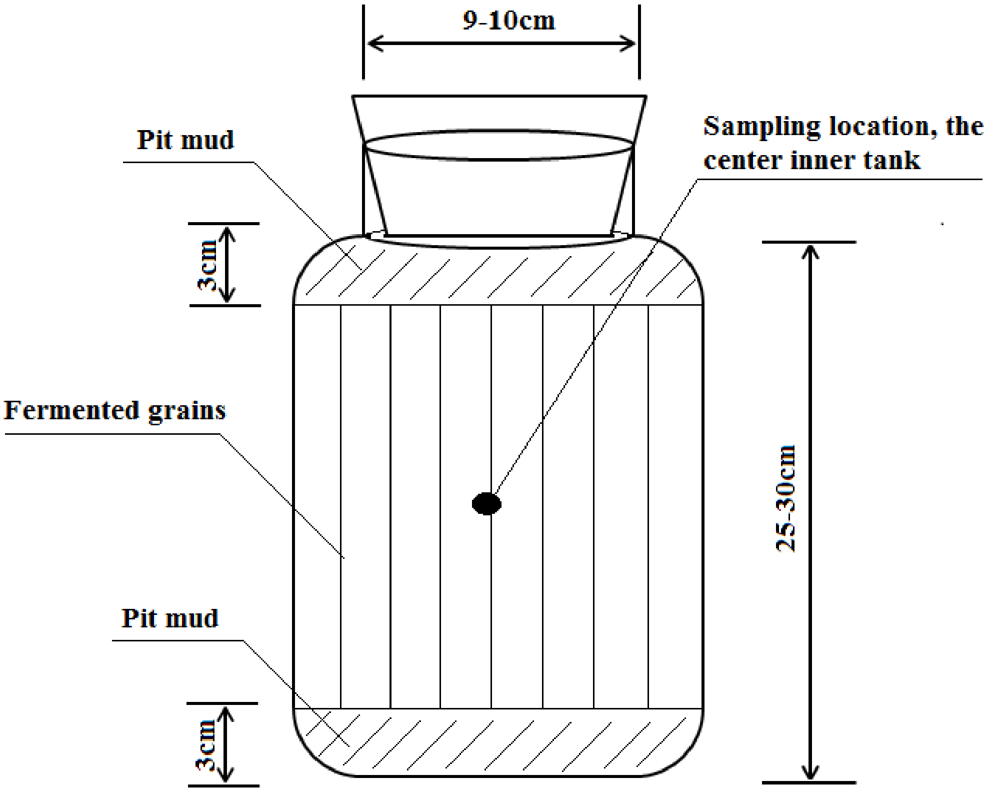

Fig S2. Diagram of glass bottles used for simulating fermentation.

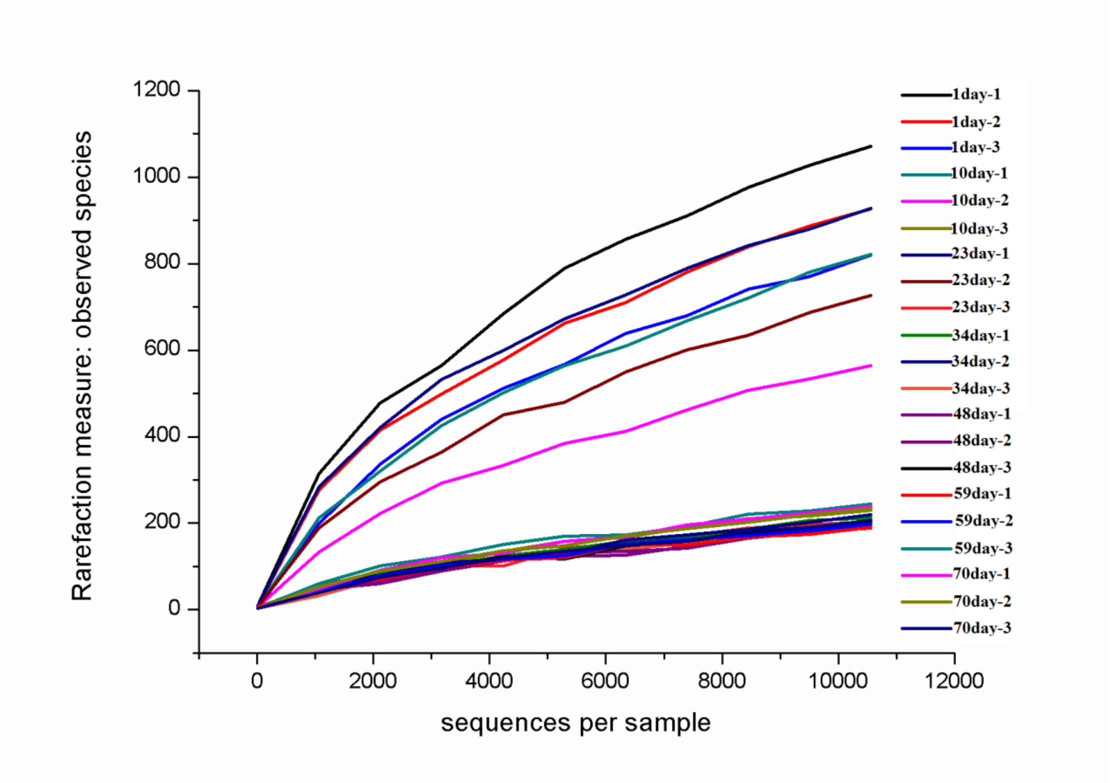

Fig S3. The rarefaction curves of sequencing depth.

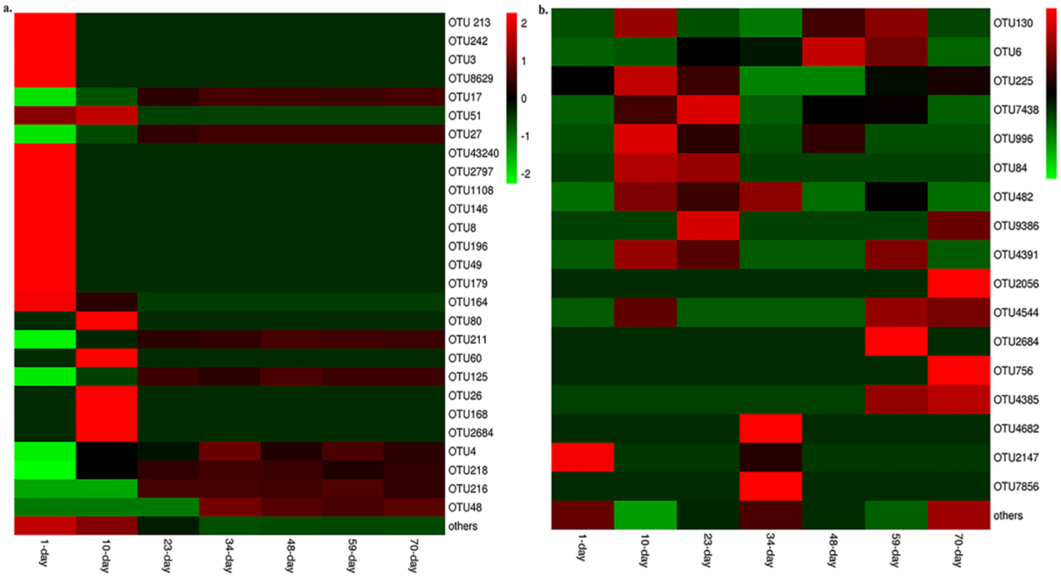

Fig S4. Heatmaps of prokaryotic (a) and eukaryotic (b) communities.

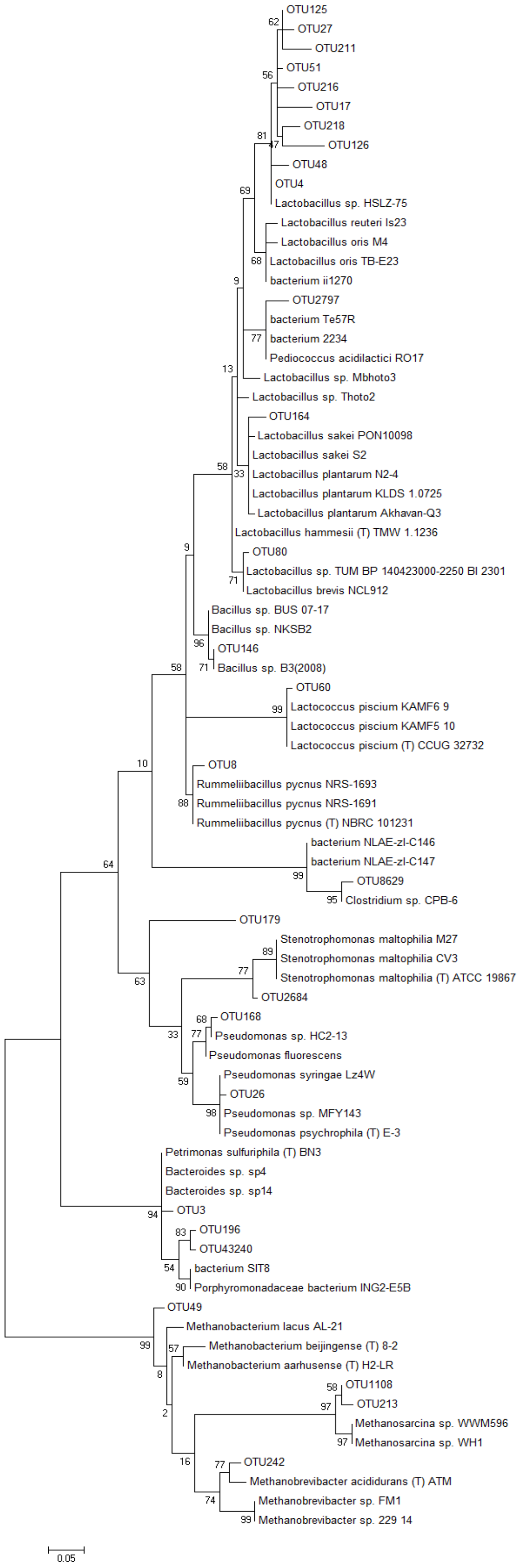

Fig S5. Phylogenetic analysis (maximum likelihood algorithm) result of prokaryotic communities.

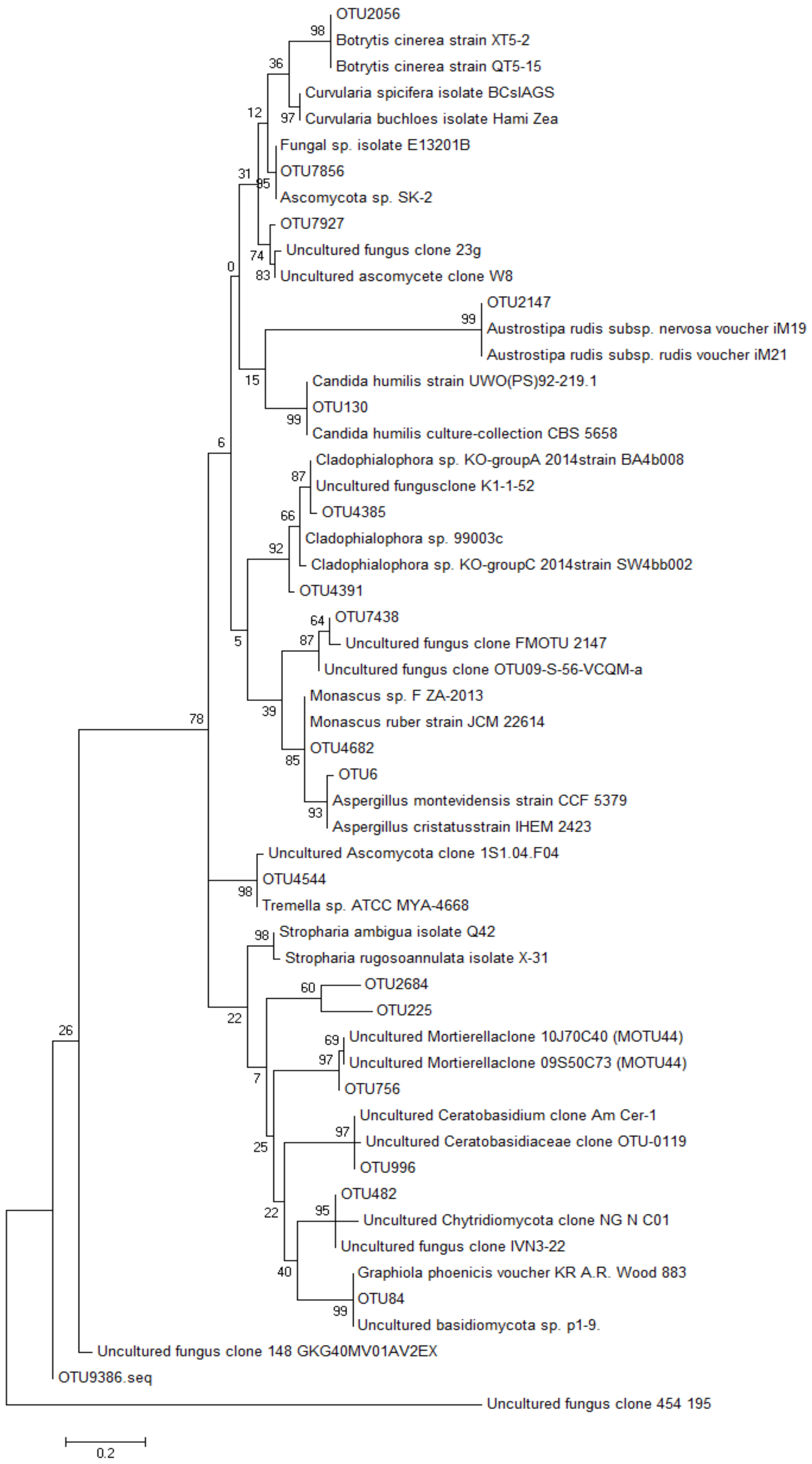

Fig S6. Phylogenetic analysis (maximum likelihood algorithm) result of eukaryotic communities.

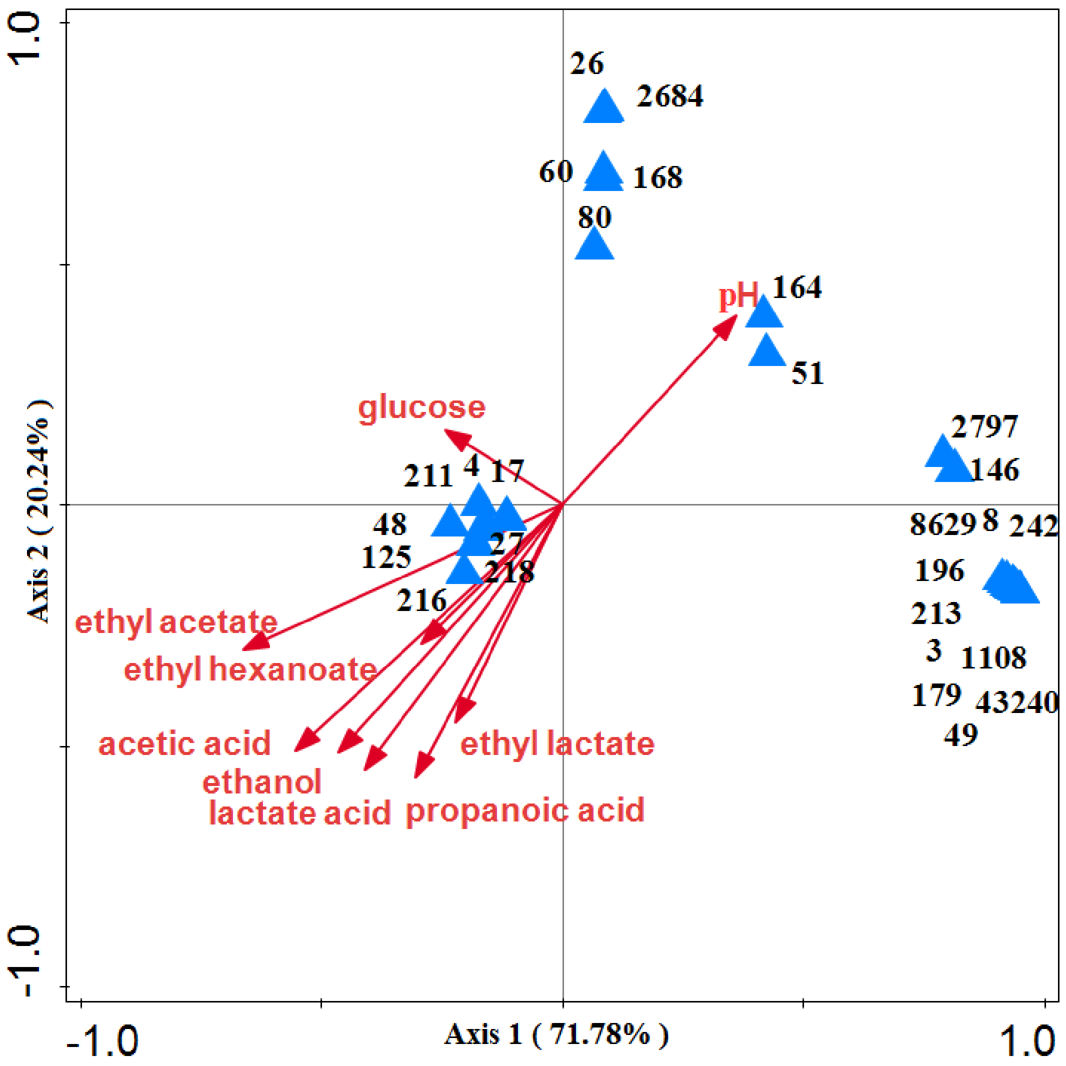

Fig S7. Canonical correspondence analysis (CCA) of prokaryotic OTUs and flavoring chemicals.

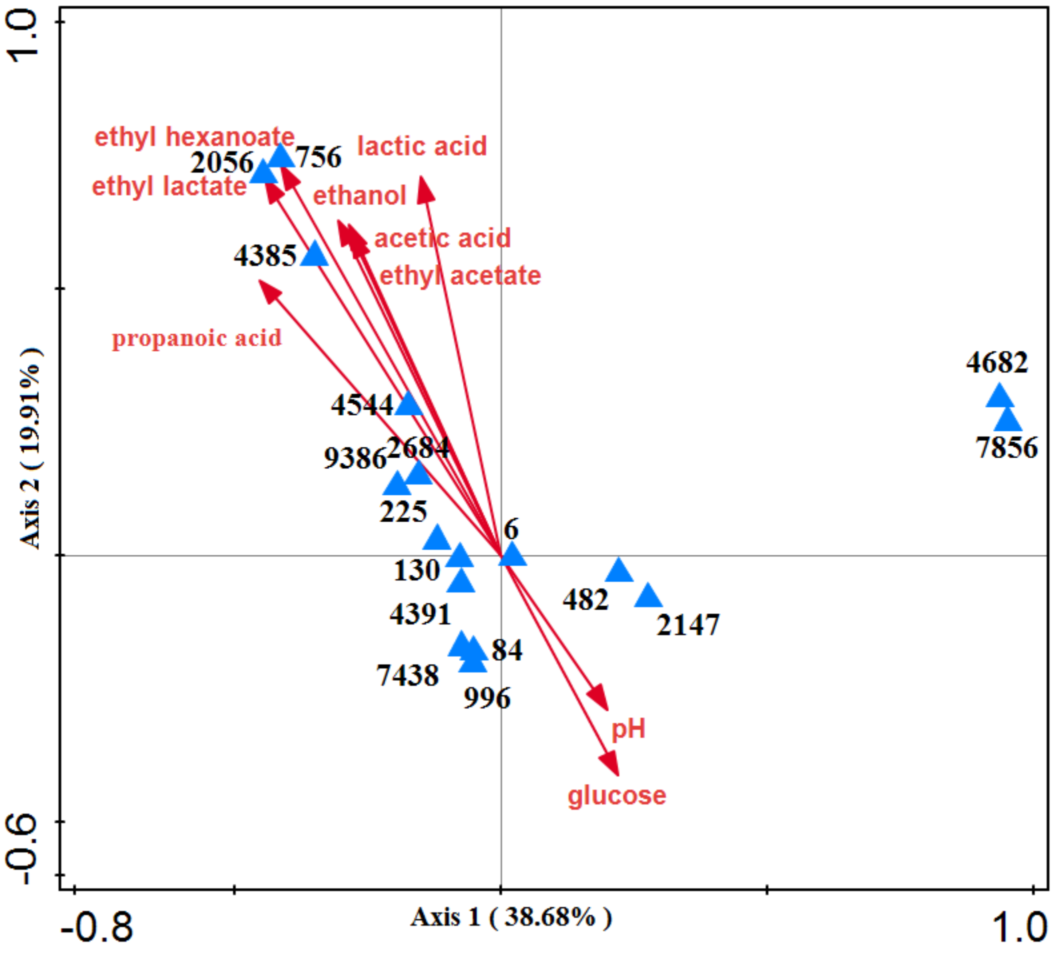

Fig S8. Canonical correspondence analysis (CCA) of eukaryotic OTUs and flavoring chemicals.
