# Supplementary material for: The process-related dynamics of microbial community during a simulated fermentation of Chinese strong-flavored liquor
Source: BMC Microbiol. 2017 Sep 15;17:196. doi: 10.1186/s12866-017-1106-3 (PMC5603089; doi:10.1186/s12866-017-1106-3)
Supplement: Supplementary file 2 — Good coverages of prokaryotic Sequencing (16S rRNA gene). Table S2. Good coverages of eukaryotic Sequencing (ITS gene). Table S3. The OTU BLAST result based on 16S rRNA gene. Table S4. The OTU BLAST result based on ITS region. (PDF 309 kb) [file 12866_2017_1106_MOESM2_ESM.pdf]

Table S1. Good coverags of 16s rRNA gene Sequencing

Sample NO.	Goods coverages
1-1	0.956
1-2	0.962
1-3	0.964
10-1	0.958
10-2	0.971
10-3	0.987
23-1	0.963
23-2	0.965
23-3	0.988
34-1	0.988
34-2	0.986
34-3	0.988
48-1	0.988
48-2	0.988
48-3	0.987
59-1	0.990
59-2	0.988
59-3	0.986
70-1	0.986
70-2	0.986
70-3	0.988

Table S2. Good coverags of ITS region Sequencing

Sample NO.	Good coverages
1-1	0.944
1-2	0.941
1-3	0.960
10-1	0.965
10-2	0.953
10-3	0.914
23-1	0.924
23-2	0.906
23-3	0.942
34-1	0.952
34-2	0.947
34-3	0.944
48-1	0.952
48-2	0.901
48-3	0.931
59-1	0.972
59-2	0.979
59-3	0.974
70-1	0.968
70-2	0.968
70-3	0.963

Table S3. OTU BLAST result based on 16s rRNA gene

OTU NO.	1-day	10-day	23-day	34-day	48-day	59-day	70-day	Reference strains	GeneBank NO.	Identity (%)
**213**	16.58% ±0.01	TR	TR	TR	TR	TR	TR	***Methanosarcina sp*. WH1**	CP009504	96.7%
**242**	5.74% ±0.02	TR	TR	TR	TR	TR	TR	***Methanobrevibacter acididurans(T)*;ATM**	AF242652	97.1%
**3**	4.25% ±0.01	TR	TR	TR	TR	TR	TR	***Petrimonas sulfuriphila* (T) BN3**	NR_042987	99%
**8629**	4.25% ±0.03	TR	TR	TR	TR	TR	TR	***NLAE－zl－C146***	JQ607884	97.1%
**17**	5.37% ±0.04	23.98% ±0.01	41.92% ±0.04	45.63% ±0.02	44.89% ±0.02	43.85% ±0.02	45.19% ±0.02	***Lactobacillus sakei*; S2**	AB470237	90.8%
**51**	1.88% ±0.00	2.44% ±0.01	TR	TR	TR	TR	TR	***Lactobacillu reuteri*; ls23**	EU381123	93.0%
**27**	3.69% ±0.03	16.46% ±0.01	26.82% ±0.04	28.2% ±0.02	28.16% ±0.02	28.11% ±0.04	28.07% ±0.01	***Lactobacillus oris*; TB－E23**	AB425929	92.5%
**43240**	1.54% ±0.00	TR	TR	TR	TR	TR	TR	***Porphyromonadaceae bacterium* ING2－E5B**	LN515532	97.6%
**2797**	2.28% ±0.01	TR	TR	TR	TR	TR	TR	***Pediococcus acidilactici*; RO17**	AF515229	98.1%
**1108**	1.36% ±0.00	TR	TR	TR	TR	TR	TR	***Methanosarcina sp*. WH1**	CP009503	95.2%
**146**	1.49% ±0.00	TR	TR	TR	TR	TR	TR	***Bacillus sp*. NKSB2**	AB305277	99.8%
**8**	1.67% ±0.01	TR	TR	TR	TR	TR	TR	***Rummeliibacillus pycnus*; NRS－1691**	AF169531	97.8%
**196**	1.31% ±0.00	TR	TR	TR	TR	TR	TR	***Porphyromonadaceae bacterium* ING2－E5B**	LN515532	97.1%
**49**	1.31% ±0.00	TR	TR	TR	TR	TR	TR	***Methanobacterium aarhusense(T)*;H2－LR**	AY386124	93.8%
**179**	1.93% ±0.01	TR	TR	TR	TR	TR	TR	***Alcaligenes sp*. BBTR16**	EF471233	98.5%
**164**	3.42% ±0.03	1.18% ±0.00	TR	TR	TR	TR	TR	***plantarum*; KLDS 1.0725**	EU626010	99.0%
**80**	TR	2.64% ±0.02	TR	TR	TR	TR	TR	***Lactobacillus brevis*; NCL912**	JF763842	99.5%
**211**	TR	5.81% ±0.01	8.09% ±0.01	8.30% ±0.01	8.83% ±0.00	8.74% ±0.01	8.64% ±0.01	***Lactobacillus sakei*; PON10098**	KC416999	91.8%
**60**	TR	2.08% ±0.01	TR	TR	TR	TR	TR	***Lactococcus piscium (T)*; CCUG 32732**	DQ343754	99.0%

**125**	TR	3.56% ±0.01	6.13% ±0.00	5.75% ±0.00	6.40% ±0.00	6.09% ±0.00	6.12% ±0.00	***Lactobacillus reuteri*; ls23**	KT783533	93.5%
**26**	TR	1.61% ±0.01	TR	TR	TR	TR	TR	***Pseudomonas psychrophila (T)*; E－3**	AB041885	99.3%
**168**	TR	2.10% ±0.01	TR	TR	TR	TR	TR	***Pseudomonas fluorescens***	AY472116	99.8%
**2684**	TR	1.76% ±0.01	TR	TR	TR	TR	TR	***Stenotrophomonas maltophilia (T)*; ATCC 19867**	AB021405	98.1%
**4**	TR	1.51% ±0.01	1.41% ±0.01	2.17% ±0.00	1.73% ±0.01	2.00% ±0.01	1.78% ±0.00	***Lactobacillus reuteri*; ls23**	EU381123	94.2%
**218**	TR	1.15% ±0.00	1.38% ±0.00	1.47% ±0.00	1.44% ±0.00	1.33% ±0.00	1.38% ±0.00	***Lactobacillus reuteri*; ls23**	EU381123	92.0%
**216**	TR	TR	1.69% ±0.00	1.66% ±0.00	1.65% ±0.00	1.74% ±0.00	1.53% ±0.00	***Lactobacillus oris*; TB－E23**	AB425929	92.5%
**48**	TR	TR	TR	1.40% ±0.00	1.20% ±0.00	1.11% ±0.00	1.23% ±0.00	***Lactobacillus oris*; M4**	KC561132	93.3%

Table S4. OTU BLAST result based on ITS region

OTU NO.	1-day	10-day	23-day	34-day	48-day	59-day	70-day	Reference strains	GeneBank ID	Identity (%)
**6**	11.7% ±0.007	12.5% ±0.03	17.6% ±0.08	16.2% ±0.06	29.6% ±0.004	25.3% ±0.04	11.4% ±0.07	***Aspergillus montevidensis* strain CCF 5379**	LT670951	99%
								***Aspergillus cristatus,* strain IHEM 2423**	LT670938	99%
**84**	TR	4.14% ±0.02	3.7% ±0.02	TR	TR	TR	TR	***Graphiola phoenicis voucher* KR A.R. Wood 883;**	KP730059	97%
								***uncultured basidiomycota sp.* p1-9**	AF207666	98%
**130**	15.4% ±0.20	34.9% ±0.02	15.5% ±0.09	11.8% ±0.13	27.7% ±0.04	32.8% ±0.009	16.3% 0.13	***Candida humilis* strain UWO(PS)92-219.1**	AY493349	99%
								***Candida] humilis culture-collection* CBS:5658**	KY102142	99%
**225**	4.0%± 0.02	6.7%± 0.007	3.8%± 0.01	TR	TR	2.1%± 0.003	3.2%± 0.001	***Curvularia buchloes* isolate Hami_Zea**	KX139031	99%
								***Curvularia spicifera* isolate BCsIAGS**	KU379555	99%
**482**	TR	2.5%± 0.005	1.8%± 0.003	2.7%± 0.01	TR	1.2%± 0.002	TR	***Uncultured Chytridiomycota* clone NG_N_C01**	GU055563	95%
								***Uncultured fungus clone* IVN3-22**	EU516787	97%

**756**	TR	TR	TR	TR	TR	TR	1.9% ± 0.01	***Uncultured Mortierella* clone 10J70C40 (MOTU44)**	HG936572	97%
								***Uncultured Mortierella*, clone 09S50C73 (MOTU44)**	HG936569	97%
**996**	TR	8.8% ± 0.02	3.6% ± 0.01	TR	3.8% ± 0.02	TR	TR	***Uncultured Ceratobasidium clone Am_Cer-1***	KJ789940	98%
								***Uncultured Ceratobasidiaceae clone* OTU-0119**	KM247422	97%
**2056**	TR	TR	TR	TR	TR	TR	1.5% ± 0.001	***Botrytis cinerea strain* QT5-15**	KX822693	99%
								***Botrytis cinerea strain* XT5-2**	KX721051	99%
**2147**	24.8% ±0.2	TR	TR	5.2% ± 0.06	TR	TR	TR	***Austrostipa rudis subsp.* rudis voucher iM21**	JF769074	96%
								***Austrostipa rudis subsp.* nervosa voucher iM19**	KC129413	96%
**4385**	TR	TR	TR	TR	TR	TR	1.4% ± 0.004	***Uncultured fungus clone*: K1-1-52**	LC096456	98%
								***Cladophialophora sp.* KO-groupA 2014, strain BA4b008**	AB986412	98%
**4391**	TR	1.8% ± 0.002	1.3% ± 0.004	TR	TR	1.5% ± 0.003	TR	***Cladophialophora sp.* KO-groupC 2014, strain SW4bb002**	AB986414	89%

								***Cladophialophora sp*. 99003c**	EU139129	89%
**4544**	TR	1.0%± 0.00	TR	TR	TR	1.1%± 0.001	1.2%± 0.001	***Tremella sp*. ATCC MYA-4668**	KC171330	98%
								***Uncultured Ascomycota clone* 1S1.04.F04**	EF619865	97%
**4682**	TR	TR	TR	4.7%± 0.05	TR	TR	TR	***Monascus sp*. F ZA-2013**	KC756830	97%
								***Monascus ruber strain* JCM 22614**	JN942658	97%
**7438**	TR	7.2%± 0.02	14.3% ±0.04	TR	4.0%± 0.01	4.84% ±0.01	TR	***Uncultured fungus clone* OTU09-S-56-VCQM-a**	JF926930	87%
								***Uncultured clone* FMOTU_2147**	KU061787	87%
**7856**	TR	TR	TR	9.0%± 0.05	TR	TR	TR	***Ascomycota sp*. SK-2**	KT240142	99%
								***Fungal sp*. isolate E13201B**	KT995907	99%
**9386**	TR	TR	2.5%± 0.02	TR	TR	TR	1.52% ±0.004	***Uncultured fungus clone* 148_GKG40MV01AV2EX**	JQ313073	81%
								***Uncultured fungus clone* 454_195**	KM493129	80%
